# Human Cardiac Organoids: Advances and Prospects from Construction to Preclinical Drug Evaluation

**DOI:** 10.3390/cells15010007

**Published:** 2025-12-19

**Authors:** Meng Chen, Tianyi Zhang, Sheng Yang, Yiru Niu, Yiling Ge, Zaozao Chen, Juan Zhang, Yuepu Pu, Zhongze Gu, Geyu Liang

**Affiliations:** 1 Key Laboratory of Environmental Medicine Engineering, Ministry of Education, School of Public Health, Southeast University, Nanjing 210009, China; 220234019@seu.edu.cn (M.C.); tyi_zhang@163.com (T.Z.); 230198330@seu.edu.cn (S.Y.); niuyiru666@163.com (Y.N.); geyiling11@163.com (Y.G.); 101011288@seu.edu.cn (J.Z.); yppu@seu.edu.cn (Y.P.); 2 Institute of Biomedical Devices (Suzhou), Southeast University, Suzhou 215163, China; 101012282@seu.edu.cn; 3 State Key Laboratory of Bioelectronics, School of Biological Science and Medical Engineering, Southeast University, Nanjing 210096, China

**Keywords:** cardiac organoids, preclinical studies, drug evaluation, pathological models

## Abstract

**Highlights:**

**What are the main findings?**

- This review proposes an integrated research framework for human cardiac organoids (hCOs) encompassing construction technology, pathological modeling, and application evaluation, and summarizes three pathological model construction strategies: patient-specific, gene-edited, and microenvironment-modulated.
- hCOs demonstrate superior sensitivity and accuracy in predicting drug-induced cardiotoxicity compared to traditional models, particularly in early warning, metabolism-related safety evaluation, and personalized drug response assessment.

**What are the implications of the main findings?**

- hCOs provide highly faithful disease models and platforms for individualized drug efficacy evaluation and offer valuable tools for studying complex cardiovascular diseases and rare disorders.
- Supported by a regulatory landscape increasingly favoring non-animal testing, hCOs, coupled with emerging technologies, hold promise for reshaping preclinical drug development into a more predictive paradigm.

**Abstract:**

Drug-induced cardiotoxicity (DICT) severely hampers drug development and threatens patient safety. Together with the growing global burden of cardiovascular disease, there is an urgent need to establish more predictive preclinical models. Recently, human pluripotent stem cell-derived cardiac organoids (hCOs) have emerged as a promising three-dimensional in vitro model, achieving significant progress in simulating the complex structure and function of the human heart. However, existing reviews predominantly focus on technical construction or specific applications, lacking an integrated discussion of pathological model construction and their use under evolving regulatory frameworks. This review distinguishes itself by proposing a novel, holistic framework that bridges “construction technology,” “pathological modeling,” and “application evaluation.” We systematically categorize and summarize three major strategies for building hCO-based pathological models: patient-specific, gene-edited, and microenvironment-modulated approaches. Furthermore, we highlight the unique advantages of hCOs in preclinical drug assessment and detail their cutting-edge applications in early DICT warning, metabolism-related safety evaluation, and personalized drug evaluation. Finally, we address current challenges, including maturation and standardization, and outline future directions involving integration with organ-on-a-chip technology and artificial intelligence. This review aims to provide a theoretical foundation and roadmap toward more reliable and human-relevant drug development paradigms.

## 1. Introduction

In the United States, the development and market launch of a new drug typically requires 12 to 15 years [1], with an estimated average cost exceeding $1 billion [2]. Unfortunately, about one-third of new drugs fail in clinical trials because of unexpected cardiotoxicity [3]. Additionally, about 10–14% of all drug withdrawals are due to drug-induced cardiotoxicity (DICT) [4]. These result in substantial financial losses and pose potential risks to patients. It has been demonstrated that currently available antiarrhythmic drugs and non-cardiac medications, including antihistamines, antipsychotics, and anti-infectives, have been implicated in clinical settings in inducing cardiomyocytes (CMs) death, arrhythmias, torsades de pointes, and heart failure, among other cardiovascular complications. These adverse effects have been shown to have a significant impact on patient prognosis and may even be life-threatening [5,6]. In particular, cardiotoxicity frequently renders anti-cancer drugs ineffective for many cancer patients [7], and it has emerged as the second leading cause of morbidity and mortality [8]. Moreover, in view of the mounting global burden of cardiovascular disease (CVDs) (projections indicate that this will claim 35.6 million lives per annum by 2050) and the global trend towards aging, there is an urgent need to accelerate the development of effective pharmaceuticals [9]. Consequently, the evaluation of drugs in preclinical phases appears to be of crucial significance, which can result in the early elimination of unsafe drugs, the optimization of the selection of drug candidates, and the reduction in risks of attrition and unnecessary resource expenditures in later stages [10].

Developing precise and reliable drug evaluation models or methods represents a pressing issue in drug evaluation and toxicological research. Primary human CMs demonstrate pharmacological responses that closely recapitulate in vivo human cardiac physiology, making them a superior model for preclinical drug evaluation [11]. However, their limited availability, inability to proliferate, and rapid dedifferentiation in vitro are significant drawbacks [12,13]. Traditional models (animal models and two-dimensional (2D) cell cultures) have been extensively utilized in drug development and preclinical studies. However, according to statistics, approximately 90% of candidate drugs fail in early clinical trials, which is partly attributed to the over-reliance on animal models in preclinical research [14]. Animal models are often limited by low throughput, time-consuming processes, relatively high costs, and ethical issues. Moreover, they exhibit species-specific differences from humans in terms of molecular and physiological characteristics, overall heart structure, and cellular responses to pathological stress [11,15,16]. For example, the proportion of cardiomyocytes in mouse hearts is roughly double that of human hearts (50% vs. 20–30%) [17], mouse heart rates are approximately nine times faster than those of humans, and electrocardiogram action potential durations (APDs) are only 1/5 to 1/10 of those in humans [18]. Additionally, the distribution and expression of α-myosin heavy chain (α-MHC also known as MYH6) and β-myosin heavy chains (β-MHC, also known as MYH7), myosin light chain 2a (MLC2a also known as MYL7), myosin light chain 2v (MLC2v also known as MYL2), and signal-regulatory protein alpha (SIRPA) differ in humans compared to mice, leading to a 10-fold higher tolerance in mice for 37% of drugs compared to humans [19]. Numerous studies in the literature have demonstrated that many candidate drugs that appear safe in preclinical 2D cell cultures and animal models exhibit unacceptable toxicity when ultimately tested in humans [20,21]. Recently, the emergence of stem cell-derived three-dimensional (3D) cultures, known as human organoids, has garnered considerable attention as a potential breakthrough in overcoming existing limitations [22].

Human organoids faithfully recapitulate native organs in vivo, bridging the gap between 2D cell cultures and animal models [23]. Their major advantage over animal models is the ability to overcome species-specific differences. Human cardiac organoids (hCOs) exhibit robust proliferative capacity, genomic stability, and precise recapitulation of intercellular interactions and complex pathophysiological processes. These features, together with their superior mimicry of human cardiac electrophysiological complexity and in vivo drug responses, provide an accurate, robust, and human-relevant platform for cardiovascular disease research, drug discovery, and cardiotoxicity testing [24,25,26].

Over the past five years, the field of hCOs has experienced rapid development, with related reviews advancing progress from diverse perspectives. However, as shown in Table 1, existing research predominantly focuses on specific aspects: some emphasize constructing techniques and maturation strategies [27,28,29]; others concentrate on particular applications, such as evaluating cardiotoxicity of anti-cancer drugs [8] or modeling cardiac development [30]; while broader overviews discuss organoid technology in general without delving into the specific challenges and opportunities of preclinical drug evaluation [31]. In addition, recent reviews have begun to explore emerging directions such as inter-organ interactions [32] and advanced assessment technologies [27,30].

Currently, there remains a lack of systematic discourse that effectively integrates organoid model construction methods with preclinical drug evaluation practices within the rapidly evolving regulatory landscape. This review aims to fill this gap by proposing a holistic framework that converges technology, models, and applications. We systematically summarize for the first time the three major strategies for constructing hCO pathological models (patient-derived, gene-edited, and microenvironment-regulated), establish a complete translational pathway from technical construction to drug evaluation, and position the value of hCOs within new regulatory contexts, highlighting their crucial role in enhancing the human relevance of preclinical drug evaluation. By offering this systematic, integrated perspective, this review aims to provide timely and comprehensive guidance for leveraging hCOs to advance drug development and safety evaluation.

## 2. Construction of hCOs

Advances in stem cell technology [33] have facilitated the differentiation of human pluripotent stem cells (hPSCs), encompassing both human embryonic stem cells (hESCs) [34] and human induced pluripotent stem cells (hiPSCs) [35], into various types of cardiovascular cells, such as cardiomyocytes (CMs), endothelial cells (ECs), cardiac fibroblasts (CFs), and epicardial cells (EpiCs). Recent studies have demonstrated that hPSCs have the capacity to be differentiated into 3D cardiac tissue, also known as hCOs. This finding has the potential to overcome the limitations of traditional 2D cultures and has been shown to demonstrate significant advantages in recapitulating human-specific cardiac responses, thus indicating great research potential [26,36,37]. hCOs are widely used for studying heart development, disease modeling, drug screening, and toxicity assessment, and they also hold great promise for precision medicine and regenerative medicine [38]. The successful construction of complex, stable, and reproducible hCOs is essential for all these applications, yet this step still faces significant challenges.

**Figure 1:**
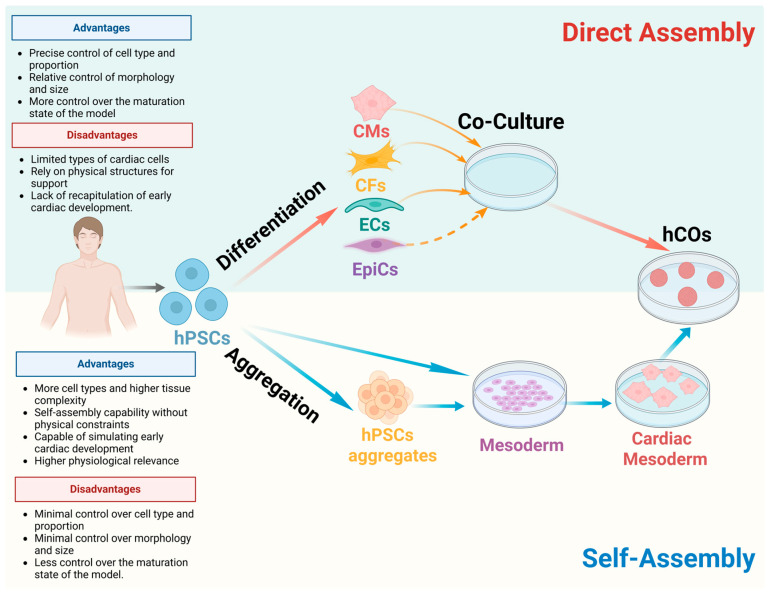
Two strategies for constructing hCOs and their respective advantages and disadvantages. The upper section illustrates the construction process and the advantages and disadvantages of the Direct Assembly strategy, while the lower section depicts those of the Self-Assembly strategy. The Direct Assembly strategy relies on co-culturing diverse hPSC-derived cardiac constituent cells—such as CMs, CFs, ECs, and EpiCs—on designated supports to generate a predefined 3D architecture. The Self-Assembly strategy recapitulates the natural principles of embryonic heart development, inducing hPSCs or their aggregates to self-organize and sequentially differentiate into mesoderm, cardiac mesoderm, and ultimately spherical 3D structures. hPSCs, human pluripotent stem cells; CMs, cardiomyocytes; ECs, endothelial cells; CFs, cardiac fibroblasts; EpiCs, epicardial cells; hCOs, human cardiac organoids.

The complexity of the human heart structure and function has resulted in limited advancements in hCOs. However, there has been an increasing popularity of hCOs in recent years [28,29,39]. With the development and application of hPSCs, there are currently two different generic schemes for generating hCOs—Direct Assembly and Self-Assembly. As shown in Figure 1, a comparison is presented between the two construction strategies, with a summary of their respective advantages and disadvantages.

### 2.1. Directed-Assembly hCOs

Directed-assembly hCOs are 3D cardiac tissues generated by the controlled combination of distinct cardiac cell types. Based on the presence or absence of external supports, direct assembly strategies can be roughly categorized into two groups: scaffold-free suspension culture and scaffold-supported culture.

In the scaffold-free suspension culture approach, cell aggregates are cultured in non-adherent round- or U-bottomed microwells to form 3D cardiac microtissues that are capable of spontaneous contraction. Studies by Giacomelli et al. [40,41] and Archer et al. [42] have demonstrated the utility of such hiPSC-derived cardiac microtissues in disease modeling and drug testing. Recent advances in this scaffold-free strategy are reflected in highly complex integrative models. For example, Yang et al. [43] developed vascularized and chambered cardiac organoids (vcCOs) in 2024. This model employs a scaffold-free, modular approach: Researchers used hiPSC-CMs to encapsulate vascular spheroids differentiated from hiPSCs. Subsequently, further vascularization of the peripheral myocardium occurred, thereby inducing the formation of vcCOs. This highlights the robust capacity and flexibility of the direct assembly strategy in constructing complex physiological microstructures.

The scaffold-supported directed-assembly hCOs [39,44,45,46,47,48,49,50,51] are also commonly referred to as human engineered heart tissues (hEHTs). In general, the formation of a predesigned 3D structure is achieved through the co-culture of a variety of hPSC-derived cardiac constitutive cells on different supports. Variations in this construction method are observed across differentiation stimulation, cell subtype specification, extracellular matrix composition, and supporting materials. Mills et al. [39,44,45] successfully constructed hCOs by mixing hPSC-derived cardiomyocytes (hPSC-CMs) with stromal cells at a 7:3 ratio, combined with collagen I and Matrigel in a well insert equipped with two elastomeric posts (the Heart-Dyno microtissue platform). Such hCOs are capable of mimicking both the cellular composition of native cardiac tissue and the function of myocardial contraction. In the study of Tiburcy et al. [46] and Ronaldson-Bouchard et al. [47], mechanical or electrical stimulation was used to promote the maturation of hPSC-CMs, thereby effectively improving the maturity of organoids. Chamber-specific hEHTs have been reported [48,49,50]. For example, Goldfracht et al. [48] produced ring-shaped hEHTs by mixing hESC-derived ventricular or atrial cells with collagen. These hEHTs exhibit distinct ventricular or atrial molecular, electrophysiological, and contractile properties. In addition, multi-chambered vascularized hCOs with a more complex structure were reported in 2023 [51], promoting our understanding of human cardiac physiology.

Directed-assembly hCOs are valuable for disease research and drug discovery, yet they have certain limitations. Current technological capabilities are inadequate in fully replicating the coexistence of diverse cell types observed in the native heart. There are discrepancies in cell ratios and densities. Moreover, the precise spatial arrangement of cells, complex interaction patterns, and the intricate regulation of morphological and physiological development in the native heart are difficult to accurately reproduce in directed-assembly hCOs [29] (Figure 1).

### 2.2. Self-Assembling hCOs

The construction of self-assembling hCOs is based on the simulation of the natural laws of embryonic heart development. In this process, hPSCs or their aggregates are induced to self-organize and differentiate into spherical 3D structures through precise regulation of key signaling pathways such as WNT, BMP, TGFβ, and FGF. The capacity of self-assembling hCOs to derive cardiac cell lineages, express gene characteristics corresponding to various stages of development in vivo, and gradually acquire the morphology and function of the heart, is well documented. Since 2021, the technology of self-assembling hCOs has made great progress, and many models have appeared, including heart-forming organoids (HFOs) [52,53], human heart organoids [54], Cardioids [55], and multi-chamber cardioids [56]. These models use hPSCs or their aggregates as a point of departure, with the occasional incorporation of hPSCs aggregates into matrigel. By using a two-step or three-step WNT signaling regulatory program (two-step: activation-inhibition, three-step: activation-inhibition-activation), complex and highly ordered 3D hCOs are constructed. In 2021, Drakhlis et al. [52,53] successfully generated HFOs using a two-step WNT signaling regulatory program, which has a high similarity to the human embryonic heart at the morphological and transcriptional levels and can reproduce many important aspects of heart development. In 2024, the team made a further breakthrough with the development of the first blood-generating HFO [57]. With a haemogenic endothelial layer containing diverse hematopoietic derivatives and hematopoietic progenitors, blood-generating HFO can simulate the cooperative development of the heart and blood system, offering a new tool for studying cardiovascular-blood interactions. In addition, Mendjan’s team successfully produced the first multi-chamber cardioids model [56] in 2023. This model offers a vivid depiction of the interaction and coordinated contraction between various cardiac compartments. In addition, it elucidates the effects of gene mutations, drugs, and environmental factors on cardiac development. This significantly promotes the development and application of chamber-specific hCOs.

In comparison with directed-assembly methods, self-assembly methods facilitate a more comprehensive study of cardiac development, allowing for diversity in organoid models. However, this approach also results in variations in overall morphology and cell proportion, as well as reduced control over the maturation state of the model (Figure 1).

Despite recent advances in hCO construction techniques, they still face common challenges, including structural and functional immaturity, poor batch-to-batch reproducibility, lack of functional vascular networks, and insufficient long-term culture stability. These limitations constrain their reliability and scalability in modeling adult cardiac physiology and pathology. Future efforts should focus on optimizing vascularization strategies, standardizing cell sources and culture systems, and integrating interdisciplinary approaches to enhance physiological relevance, thereby advancing hCOs toward more predictive pharmacological and disease models [27].

## 3. Construction Strategies of Cardiac Pathological Models

The controllability of hCOs, coupled with their capacity to accurately reproduce structural and functional phenotypes, has led to their emergence as a superior tool for constructing cardiac pathological models. These models can effectively simulate the pathological characteristics of various heart diseases, including congenital heart disease (CHD), myocardial infarction (MI), heart failure, and arrhythmias [58]. Currently, researchers primarily employ gene editing technologies to introduce or knock out specific genes in hCOs to construct disease-specific hCOs or utilize patient-derived hPSCs to construct patient-specific hCOs, thereby simulating the onset of CHD and genetic diseases. In addition, the pathological model of acquired heart disease can be established through physical injury, chemical stimulation, and viral infection. These construction strategies provide high-fidelity model platforms for research into heart disease, exploration of precision medicine, and assessment of drug toxicity. In this section, three strategies for constructing hCO pathological models will be discussed (Table 2).

### 3.1. Based on Patient-Derived hPSCs

hPSCs obtained by reprogramming of somatic cells from a specific patient can be directly differentiated into hCOs with natural pathological features, that is, patient-derived hCOs. In comparison with conventional models, patient-derived hCOs maintain the distinctive patient-specific genetic background, incorporating particular genetic variations. This capability provides a valuable platform for modeling complex human diseases such as hypertrophic cardiomyopathy (HCM) and dilated cardiomyopathy (DCM), which have been challenging to study with existing animal models [59]. Among patient-derived hCOs, several directed-assembly hCOs have been used to model various types of inherited myocardial injury, including HCM [60,61,62,63,64], DCM [65,66,67], restrictive cardiomyopathy (RCM) [68], arrhythmogenic cardiomyopathy (ACM) [69], and arrhythmogenic right ventricular cardiomyopathy (ARVC) [70]. For instance, patient-derived hPSCs carrying mutations in α-actin 2 (ACTN2) [60], MYH7 [61], MYBPC3 [62], BRAF [63], and PRKAG2 [64] were used to construct directed-assembly hCOs, respectively. A series of pathological features of HCM, such as hypertrophy, contractile dysfunction, and prolonged APD, are summarized in Table 2. Using directed-assembly hCOs, researchers have successfully mimicked the pathological features of arrhythmias caused by genetic variants, such as prolonged APD [71]. In addition, self-assembling hCOs have been utilized by a few groups to explore the pathological mechanism of cardiomyopathy, including HCM [72] and Duchenne muscular dystrophy (DMD) [73,74]. These studies have provided innovative tools and methods for a deeper understanding of these complex diseases. To establish a clear causal link between gene mutations and disease phenotypes, it is essential to rule out interference from other genetic variations. For this purpose, some of these studies [60,64,66,67,68,73] have employed gene-editing tools such as CRISPR/Cas9 to create control cell lines—isogenic control hPSCs. These control cell lines share an identical genetic background with the patient-derived hPSCs but lack the specific pathogenic mutations. Researchers then used these cell lines to construct patient-derived hCOs and isogenic control hCOs and compared their phenotypes.

Patient-derived hCOs provide patients with personalized disease models, which not only help to predict the response of a specific patient to treatment but also can advance the development of precision medicine. Furthermore, the application of gene editing technology is imperative for the study of specific genetic effects in syngeneic backgrounds [75]. Although patient-derived hCOs hold promise for precision medicine, their limited scalability represents a major analytical bottleneck for clinical translation. First, patient-derived somatic cells can be difficult to obtain, especially in the context of rare diseases [11]. Second, establishing patient-derived hPSCs cell models is usually time-consuming and expensive. Finally, batch differences among individuals make large-scale drug efficacy comparisons complex. Therefore, some researchers have contemplated the prospect of employing gene editing of wild-type cell lines to generate disease-specific models, as an alternative to the time-consuming and costly process of constructing patient-specific disease models that may not be suitable for the intended study [12].

### 3.2. Based on Introduced Mutation or Knock-Out

The combination of genetic technologies such as CRISPR/Cas9 and organoid models provides a unique platform for the “developmental tracing” of disease mechanisms [76]. Most current research focuses on the introduction of disease-causing mutations or key gene knockout in healthy hPSCs to construct disease-specific hCOs for studying cardiac developmental diseases [52,55,56,77,78,79,80]. Self-assembling hCOs closely mimic natural cardiac development and capture heart cell heterogeneity, making them ideal for studying heart development and complex multicellular interactions [81]. The interference of gene mutation on key events of cardiac development can be observed in real time during the differentiation of self-assembling hCOs, and the dynamic regulatory network from gene mutation to pathological phenotype can be revealed. For instance, Drakhlis et al. [52] utilized NKX2.5 knockout hESCs (NKX2.5-KO hESCs) to develop heart-forming organoids (HFOs). In these HFOs, the researchers identified pathological features such as reduced cardiomyocyte adhesion, hypertrophy, and disarrangement of sarcomeres, and these heart defect phenotypes were consistent with those observed in the NKX2.5 knockout mouse (NKX2.5-KO mouse) model. In recent years, the combination of gene editing technology and hCO technology has opened up a new vision for in vitro modeling of complex CHD. For instance, hypoplastic left heart syndrome [55], Ebstein’s anomaly [77], and Noonan syndrome [79] have been effectively modeled in vitro using hCOs. In a recent study [56], Sasha Mendjan’s team combined multi-chamber cardioids with CRISPR-Cas9 technology to successfully construct a model that simulates compartment-specific defects. They knocked out the ISL1, TBX5, and FOXF1 genes, which encode cardiac transcription factors (TFs), respectively, confirming a causal relationship between mutations in these genes and compartment-specific defects. Apart from the above studies, a few studies [78,80] induce specific gene mutations to construct directed-assembly hCO pathological models for hereditary heart disease research (such as HCM, DCM, and DMD cardiomyopathy).

hCO pathological models constructed via gene editing enable precise simulation of monogenic disease mechanisms, yet they struggle to recapitulate key pathological features such as polygenic interactions, dynamic disease progression, and complex microenvironmental contexts. Future efforts should focus on integrating multi-gene editing, spatiotemporally controlled regulation, and bioengineering strategies, along with promoting cross-model validation and standardized construction, to enhance their physiological relevance and predictive power for clinical translation.

### 3.3. Based on Microenvironment Regulation

In addition to the above two construction methods, an increasing number of researchers are stimulating hCOs by physical, chemical, or biological factors to modulate their culture microenvironment to mimic the pathological process of various heart diseases. Compared with traditional animal models or gene-edited organoid construction programs, this strategy exhibits a reduced experimental period, thereby substantiating the distinctive merits of precise regulation of the metabolic microenvironment in disease modeling. For instance, Aguirre’s team cultured self-assembling hCOs in a differentiation medium that simulates pregestational diabetes (PGD) and contains high insulin and glucose to investigate the effects of PGD on cardiac development [82,83,84]. Within about 2 weeks of hCOs differentiation, the researchers detected the core pathological phenotypes of PGD-induced CHD, such as signs of cardiac hypertrophy and arrhythmia, as well as changes in glucose and lipid metabolism [82,83]. In 2023, the team further revealed the underlying mechanism of PGD-induced CHD through the pathological model of hCOs, which provided a new target for the prevention and treatment of this disease [84]. Studies have confirmed that the PGD-induced CHD core phenotype can be stably reproduced only by adjusting the glucose and insulin concentrations in the hCOs culture system. In recent years, the self-assembling hCO model has made a breakthrough in the research of myocardial ischemic diseases. The researchers successfully simulated the dynamic pathological process of MI by precisely regulating the microenvironment parameters. Among them, oxygen concentration gradient control and low temperature control became the key methodological breakthroughs. Oxygen concentration is a key factor affecting the metabolism and function of CMs. Some studies have successfully simulated the changes in cardiac structure and function after MI under the conditions of hypoxia and norepinephrine [85] or hypoxia-induced ischemia and ischemia–reperfusion (IR) injuries [86]. It was found that hCOs cultured under these conditions exhibited pathological features similar to those of MI, including metabolic changes, fibrosis, and abnormal calcium handling [85,86]. Other groups have successfully simulated the pathological features of myocardial fibrosis by cryoinjury [43,55,87].

It has been proven that stimulation of hCOs with specific drugs can create a model of cardiotoxicity [45,51,88,89,90,91,92,93]. In one study [89], doxorubicin (DOX) treatment of hCOs successfully mimicked the chemotherapy-induced cardiotoxicity. Another group [51] generated an arrhythmia model by stimulating hCOs with Mitoxantrone. This provides new insights into the understanding of chemotherapy-related cardiac injury. During the COVID-19 pandemic, several groups have used the hCO model to study the effects of the SARS-CoV-2 virus on the heart. The investigators mimicked SARS-CoV-2 infection in several ways, including stimulating hCOs with cardiac cytokine storm (which includes IFN-γ, IL-1β, and poly (I:C)) [45] or with IL-1β alone [90], as well as directly using the SARS-CoV-2 virus on hCOs [91]. These methods successfully simulated the pathological changes in heart injury in COVID-19 patients, and revealed the transcriptome, structural, and functional characteristics related to COVID-19, including inflammatory response and the damage of CMs’ structure and function. These hCO pathological models provide valuable tools for studying the effects of emerging infectious diseases on the heart. In 2025, a study [92] successfully constructed the first in vitro integrated model of Heart Failure with Preserved Ejection Fraction (HFpEF) comorbidities (obesity, diabetes, and hypertension) in hCOs through a multidimensional microenvironmental precision regulation strategy. This model provides a novel experimental platform for studying the pathological mechanisms of HFpEF and the synergistic effects of comorbidities by simulating the complex interactions among multiple factors.

While constructing pathological models through precise modulation of the microenvironment offers a flexible strategy, key analytical challenges must be addressed before their application in long-term drug screening. These include ensuring standardized stimulation conditions across different batches and verifying whether the pathological phenotype remains stable after stimulus removal (long-term stability).

**Table 2:** Construction of hCO pathological models.

Classification	Manufacture Methods of hCOs	Methods	Pathological Models	Pathological Features	Refs
Based on patient-derived hPSCs	Direct assembly	HCM patient with ACTN2 mutation	HCM	Hypertrophy, myofibrillar disarray, hypercontractility, impaired relaxation, prolonged APD	[60]
	Direct assembly	HCM patient with MYH7 mutation	HCM	Hypertrophy, CM apoptosis, contractile dysfunction	[61]
	Direct assembly	HCM patient with MYBPC3 mutation	HCM	Hypertrophy, prolonged APD, slower calcium transients, preserved twitch duration	[62]
	Direct assembly	Cardio-facio-cutaneous syndrome patient with BRAF mutation	HCM	Hypertrophy, arrhythmias, increased contraction force and relaxation, reduced duration	[63]
	Direct assembly	Cardiomyopathy patient with PRKAG2 mutation	HCM	Hypertrophy, glycogen accumulation, increased contraction force	[64]
	Self-assembly	HCM patient	HCM	Hypertrophy, arrhythmias	[72]
	Direct assembly	RCM patient with FLNC mutation	RCM	Increased passive tension, impaired relaxation velocity	[68]
	Direct assembly	DCM patient with KLHL24 mutation	DCM	Desmin degradation, tissue dilatation, impaired mitochondrial function, decreased force values, increased CM stress	[65]
	Direct assembly	DCM patient with PLN p Arg14del mutation	DCM	Impairment of the endoplasmic reticulum /mitochondria compartment, irregular beating	[66]
	Direct assembly	DCM patient with cTnT mutation	DCM	Mitochondrial oxidative phosphorylation defect, decreased amplitude of contraction, increased fibrosis	[67]
	Self-assembly	DMD patient	DMD	Reduced beat rate over time, CM deterioration, fibrosis, aberrant adipogenesis	[73]
	Self-assembly	DMD patient	DMD	Contractile dysfunction, calcium handling dysfunction	[74]
	Direct assembly	ACM patient with desmoplakin mutation	ACM	Arrhythmias, increased diastolic length, contractile shortening, reduced number of desmosomes	[69]
	Direct assembly	ARVC patient with PKP2 and DSG2 mutation	ARVC	CM apoptosis, abnormal adipogenesis, and calcium handling dysfunction	[70]
	Direct assembly	Long QT syndrome type 2 patient	Arrhythmias (genetic variants)	Prolonged APD	[71]
	Direct assembly	Catecholaminergic polymorphic ventricular tachycardia type 2 patient	Arrhythmias (genetic variants)	Arrhythmias (double peaks)	[71]
Based on introduced mutation or knock-out	Self-assembly	NKX2.5 knockout	Cardiac malformations	Decreased CM adhesion, hypertrophy, disorganized sarcomeres	[52]
	Self-assembly	HAND1 knockout	Hypoplastic left heart syndrome	Deficient cavity formation	[55]
	Self-assembly	NKX2.5 mutation	Ebstein’s anomaly	Sarcomere structure defects, lower CM differentiation efficiency	[77]
	Self-assembly	PTPN11 mutation	Noonan syndrome	Myocardial fibrosis, increased CM proliferation	[79]
	Self-assembly	ISL1 knockout	Compartment-specific defects	Delayed onset of contraction, atrial and outflowtract malformations	[56]
	Self-assembly	TBX5 knockout	Compartment-specific defects	Spontaneous beating-lacking, impaired CM differentiation	[56]
	Self-assembly	FOXF1 knockout	Compartment-specific defects	Reduced contraction, abnormal cavity formation, differentiation impairments (atrial and atrioventricular canal)	[56]
	Direct assembly	DMD mutation	DMD	Contractile and calcium transient defects	[80]
	Direct assembly	TNNT2 mutation	HCM and DCM	Hypercontractility in HCM-EHTs, hypocontractility in DCM-EHTs	[78]
Based on microenvironment regulation	Self-assembly	High insulin and high glucose	Pregestational diabetes induced CHD	Larger size, arrhythmias, decreased oxygen consumption, increased glycolysis	[82,83,84]
	Self-assembly	High glucose and high lipid	Diabetic cardiomyopathy	Oxidative stress, mitochondrial dysfunction, increased expression of cardiac injury markers, fibrosis-related genes and inflammatory cytokines	[94]
	Self-assembly	Cryoinjury	Cardiac injury	Cardiac cell death, reduced contractile function, fibrosis	[87]
	Self-assembly	Cryoinjury	Cardiac injury	Fibrosis, cardiac cell death	[55]
	Direct assembly	Hypoxia and norepinephrine	MI	Pathological calcium handling, contractile dysfunction, reduced oxygen consumption, fibrosis	[85]
	Direct assembly	Cryoinjury	MI	Myocardial injury, fibrosis, reduced calcium handling capacity	[43]
	Self-assembly	Hypoxia-induced ischemic injury and ischemic-reperfusion injury	MI and cardiac fibrosis	Cardiac cell death, disrupted sarcomere structure, reduced cardiac markers, calcium overload, defects in calcium handling, fibrosis	[86]
	Direct assembly	SARS-CoV-2 infection	COVID-19 myocarditis	CM infection, myocardial inflammation, and contractile dysfunction, CM death, sarcomere breakdown	[91]
	Self-assembly	Cardiac cytokine storm including IFN-γ, IL-1β, and poly (I:C) stimulation	Cardiac dysfunction	Increased CM apoptosis, depressed contractility, alterations in electrophysiological function, calcium handling, and sarcomere organization	[45]
	Self-assembly	IL-1β stimulation	COVID-19 acute cardiac injury	Depressed cardiac function, pathological cardiac fibrosis, thrombotic formation, vascular damage	[90]
	Self-assembly	Stimulation with inflammatory cytokines (IFN-γ, IL-1β, TNF-α)	Heart inflammation	Contractile dysfunction, fibrosis, energy metabolic disorders	[95]
	Direct assembly	Doxorubicin stimulation	Cardiotoxicity	Impaired contractile function, arrhythmias	[89]
	Direct assembly	Mitoxantrone stimulation	Arrhythmias	Irregular contractions, electrical depolarization, and oxygen oscillations	[51]
	Direct assembly	Neurohumoral overstimulation	Heart failure	Contractile dysfunction, CM hypertrophy, CM death, and N-terminal pro B-type natriuretic peptide release	[46]
	Direct assembly	Metabolites (C18:1AC) stimulation	Atrial fibrillation	Impaired mitochondrial respiration, contractile dysfunction	[93]
	Self-assembly	ET-1	Cardiac hypertrophy	Thickened chamber walls, reduced fractional shortening, increased myofibrillar disarray	[88]
	Self-assembly	Hypertension induction: AT-II and ET-1	HFpEF	Fibrosis, preserved contractile force, hypertrophy, increased ROS production, mitochondrial dysfunction, decreased ATP content, prolonged relaxation, impaired calcium handling	[92]
		Obesity-related inflammation: IL-1β and IFN-γ			
		Diabetes induction: High glucose and insulin deprivation			

hPSCs, human pluripotent stem cells; HCM, hypertrophic cardiomyopathy; APD, action potential duration; CM, cardiomyocyte; RCM, restrictive cardiomyopathy; DCM, dilated cardiomyopathy; DMD, Duchenne muscular dystrophy; ACM, arrhythmogenic cardiomyopathy; ARVC, arrhythmogenic right ventricular cardiomyopathy; EHTs, engineered heart tissues; CHD, congenital heart disease; MI, myocardial infarction; ET-1, Endothelin-1; AT-II, Angiotensin-II; HFpEF, heart failure with preserved ejection fraction.

In constructing hCO pathological models, it was found that directed-assembly hCOs and self-assembling hCOs each have unique characteristics, rendering them suitable for studying specific diseases. Self-assembling hCOs rely on the self-organizing ability of cells, and this model is more closely aligned with the biological processes of cardiac development. Consequently, they demonstrate a high level of proficiency in the field of modeling congenital structural malformations and various developmental diseases. In contrast, directed-assembly hCOs provide greater flexibility and control in modeling specific diseases by achieving precise control over cellular arrangement and tissue structure through external intervention. Three types of construction strategies covered the core needs of heart disease research from individual, gene, and microenvironment dimensions, respectively. However, each strategy exhibits notable differences in reproducibility, translational relevance, cost, and scalability, as well as suitability for specific cardiac diseases. These factors directly influence their selection in both basic research and preclinical applications. To provide a clear comparison of their core characteristics, Table 3 systematically summarizes the comparative analysis across these dimensions. As shown in Table 3, each of the three modeling strategies has distinct strengths and limitations. Patient-specific models offer significant value in precision medicine but are constrained by reproducibility and cost. Gene-edited models provide excellent controllability and reproducibility for mechanistic studies but are inadequate for modeling polygenic diseases. Microenvironment-modulated models, on the other hand, excel in cost-effectiveness and scalability, making them particularly suitable for simulating common acquired heart diseases. Therefore, when constructing pathological models, researchers need to take into account a variety of factors, including the complexity of the disease, the specific goals of the research, and resource constraints, to ensure that the constructed models most effectively support their research needs. In the future, the combination of multi-omics analysis (e.g., single-cell sequencing and spatial transcriptomes) with dynamic culture systems (e.g., organ-on-a-Chip (OoC)) will further enhance the spatiotemporal resolution of pathological models and provide a more powerful platform for mechanism analysis, treatment strategy development, and drug research in heart disease.

## 4. The Advantages and Applications of hCOs in Preclinical Drug Evaluation

**Figure 2:**
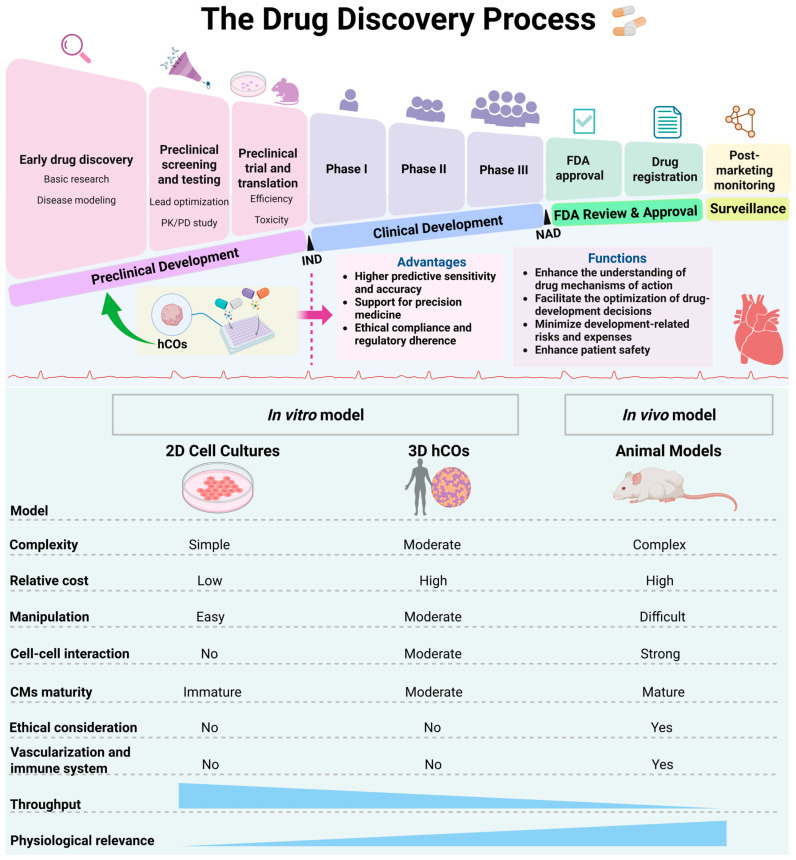
Comparison of hCOs with other models and their advantages and functions in preclinical drug evaluation. The figure systematically illustrates the integrated application and comparative value of human hCOs in drug development. The upper section presents the complete drug discovery process in a flowchart format—spanning from early-stage research and preclinical screening to clinical trials, regulatory review and approval, and post-marketing surveillance—while highlighting the key roles and advantages of hCOs in the preclinical phase. The lower section employs a comparative table to systematically contrast three major preclinical models—2D cell cultures, 3D hCOs, and animal models—across nine dimensions: complexity, cost, manipulation, cell–cell interaction, maturity, ethical consideration, vascularization and immune system, throughput, and physiological relevance. IND, Investigational New Drug; NAD, New Drug Application; hCOs, human cardiac organoids; CMs, cardiomyocytes.

As previously stated, conducting preclinical cardiac safety assessments is essential in the process of drug development. Early detection of potential cardiotoxicity through such assessments enhances patient safety, minimizes development-related risks and expenses, and supports compliance with regulatory requirements. Moreover, they facilitate the optimization of drug-development decisions and enhance the understanding of drug mechanisms of action. Presently, in the field of preclinical drug evaluation, the advantages of hCOs over traditional 2D cell models and animal models have become increasingly prominent, evident (Figure 2), resulting in their growing application.

### 4.1. Advantages of hCOs for Preclinical Drug Evaluation

In the preceding section, a comparative analysis was conducted between hCOs and 2D cell models as well as animal models (Figure 2). With the development of stem cell technology, the Comprehensive in vitro Proarrhythmia Assay (CiPA) initiative proposed by the FDA advocated the utilization of hPSC-CMs as a model for evaluating drug-induced cardiotoxicity (DICT) in 2013 [96]. Although hPSC-CMs are widely used in the preclinical research of DICT due to their superior genetic relevance [97], they exhibit immaturity in structure and function (such as impaired electrophysiology and calcium handling, compromised contractile function, and underdeveloped T-tubules) [98], as well as inaccurate drug responses when cultured in 2D conditions, collectively limiting their ability to model CVDs and drug responses accurately [99]. Research has demonstrated that organoid models, with their unique advantages, can effectively identify adverse drug reactions and help reduce the morbidity and mortality of patients in clinical trials [100]. In this section, the three distinctive advantages of hCOs in preclinical drug evaluation are summarized (Figure 2).

#### 4.1.1. Higher Predictive Sensitivity and Accuracy

In the context of preclinical toxicity assessments, hCOs have exhibited superior predictive sensitivity and accuracy, suggesting their potential as a promising platform for early cardiotoxicity screening. Compared to traditional methods, hCOs can reflect drug toxicity in the complex cardiac microenvironment, covering impacts on diverse cardiac cells and intercellular drug response crosstalk [100]. Also, by real-time monitoring of hCOs’ contractile function, calcium signaling, and metabolic status, drug toxicity signals could be captured more promptly and sensitively, thereby providing early warnings of potential cardiac safety risks. For instance, when monitoring hCOs exposed to cardiotoxic drugs (like Astemizole, Cisapride), an increase in time-dose-dependent cell death was observed, accompanied by a progressive decrease in ATP activity. Conversely, changes in 2D-cultured CMs were comparatively small, suggesting that hCOs are more sensitive in detecting cardiotoxicity [101]. A series of experiments was conducted on a wide range of clinically relevant compounds, including antibiotics, anti-diabetic drugs, and anti-cancer drugs. The results indicated that the detection method based on functional contractility exhibited enhanced sensitivity compared to conventional activity detection in predicting DICT, demonstrating strong concordance with clinical observations [102].

A study [44] revealed that some compounds that could promote CMs’ proliferation without affecting contractile function in the 2D system failed to reproduce the same effect in 3D hCOs. This suggests that 3D hCOs may have higher predictive accuracy in functional screening, thereby helping to eliminate false positives in traditional 2D culture. It was important to note that the emergence of a series of chamber-specific hCOs is particularly conducive to studying the chamber-specific toxicity of drugs, accurately identifying the differences in drug toxicity targeting, and thus further improving the accuracy of drug assessment [103].

#### 4.1.2. Support for Precision Medicine

hCOs also demonstrate unique advantages in the field of personalized drug assessment and are expected to achieve precise drug evaluation. The variability in patients’ responses to therapeutic drugs and their susceptibility to DICT are primarily attributable to patients’ risk factors and genetic susceptibility [104,105]. However, in the current field of drug research and development, there is a lack of individualized models to reflect patient-specific drug responses [106]. hCOs constructed based on hiPSCs technology could completely preserve individual genetic information and accurately simulate drug responses under pathological conditions, which are used to evaluate patient-specific DICT, thereby significantly improving the accuracy and safety of individualized treatment plans [31,59]. Moreover, hCOs hold the potential to compensate for the deficiencies of research models for rare diseases. Children suffering from rare diseases have a relatively short survival period (30% of them do not survive beyond the age of five), while the preclinical research period of traditional animal models is as long as 6 to 8 years [74]. The construction of human organoids based on patient cells has the potential to extend the treatment time, thereby generating new hope for precision medicine and rare diseases. With technological advancements, such personalized evaluation can mitigate drug-induced adverse reactions caused by individual differences, improve pharmacotherapy safety and efficacy, and lay the foundation for the realization of precision medicine in the true sense.

#### 4.1.3. Ethical Compliance and Regulatory Adherence

In the domain of preclinical drug safety evaluation, hCOs also demonstrate remarkable advantages regarding ethical and regulatory compliance. The advent of human organoid technology has emerged as a novel approach to address the ethical challenges inherent in drug research for children and pregnant women. There are significant differences in drug responses between children and adults. Nevertheless, due to ethical considerations, related research is limited. The organoids constructed using hPSC technology have the potential to facilitate the study of drug responses in children, providing a scientific foundation for the development of safer medication regimens for pediatric patients [107]. Similarly, research on drug safety during pregnancy is restricted by ethical constraints, while organoids can serve as an alternative approach to fill this research gap and show broad application prospects [14]. Furthermore, the wide application of organoids contributes to a reduction in reliance on animal experimentation, a practice which is in accordance with ethical principles and current policies and regulations.

As an internationally recognized and most widely applicable industry guideline, the International Council for Harmonisation of Technical Requirements for Pharmaceuticals for Human Use (ICH) pointed out in “E14/S7B Q&As” [108] released in 2022 that hCOs can be used as an alternative model for preclinical assessment of the risk of drug-induced QT interval prolongation. Immediately following, in the following year’s “S12: Nonclinical Biodistribution Considerations for Gene Therapy Products” [109], the application of alternative methods, including organoids, was mentioned again. These technical guidelines are expected to undergo continuous refinement in the future, providing a foundation for a globally unified methodological framework and thereby facilitating the advancement of organoid technology in biomedical research and clinical applications. Meanwhile, international regulatory agencies such as the FDA are gradually recognizing and actively encouraging the use of in vitro models like organoids for non-clinical safety assessment of drugs, thereby breaking through the limitations existing in traditional animal models. In 2017, the FDA released the “Predictive Toxicology Roadmap” [110], proposing the strategic goal of reducing animal experiments. In 2020, the FDA established the “Alternative Methods Working Group” [111], dedicated to promoting the wide application of emerging technologies such as organoids. In 2022, the “FDA Modernization Act 2.0” [112] was promulgated. For the first time, the requirement of mandatory animal experiments for preclinical research of new drugs was abolished at the legislative level, and it was explicitly allowed to conduct preclinical trials using New Approach Methodologies (NAMs) such as organoids and OoC. In 2024, “FDA Modernization Act 3.0” [113] mandates an update of the regulatory system to further promote the implementation of the FDA Modernization Act 2.0. As of 10 April 2025, the FDA announced a landmark policy shift [114], namely the plan to gradually reduce the reliance on animal experiments in the research and development of monoclonal antibodies and other drugs. This series of initiatives is indicative of a major transformation in the field of drug research and development. Furthermore, it paves the way for the wide application of organoid and OoC technologies. In addition, future regulatory incentive policies will play a significant role in enhancing the efficiency of technology transformation. The FDA has committed to implementing an accelerated review process for enterprises that submit high-quality non-animal data [114]. On 29 April 2025, the National Institutes of Health (NIH) announced a new plan, proposing to cut funds for animal experiment projects and instead increase investment in NAMs-based research [115]. Subsequently, on 7 July, the NIH announced an end to funding for animal-only studies [116].

The continuous upgrading of the regulatory framework has significantly enhanced the compliance value of hCOs in drug research and development, perfectly aligning with the “3R principles” advocated by modern regulatory science and the orientation towards technological innovation. Regulatory acceptance of NAMs directly depends on their key performance metrics. For example, the ICH E14/S7B Q&As guideline permits the use of hPSC-CMs for assessing QT-interval-prolongation risk, provided the models demonstrate reliable and reproducible electrophysiological characterization [108]. hCOs outperform traditional 2D models in electrophysiological maturity, 3D structural complexity, and electromechanical coupling integrity, enabling a more faithful simulation of human cardiac responses [24] and thereby better fulfilling ICH requirements for clinical relevance and predictive value.

Concurrently, the FDA underscores the need for alternative models to provide robust scientific evidence to support regulatory decision-making. This demands that the development of hCOs focus on standardization, rigorous validation, and systematic characterization. In the future, only when widely accepted standards and databases are established for critical performance indicators—such as reproducibility, predictive accuracy, and maturation status—can hCOs truly serve as alternatives to animal testing. Therefore, current advances in manufacturing processes, high-content analysis, and integration with AI represent not merely technological iterations but essential steps in driving the evolution of regulatory science.

### 4.2. Application of hCOs for Preclinical Drug Evaluation

**Figure 3:**
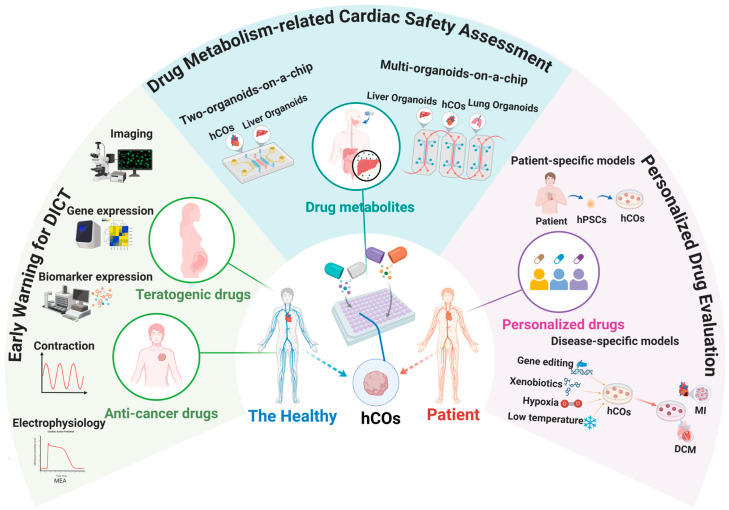
Application of hCOs for preclinical drug evaluation. The figure systematically demonstrates the multi-scenario integrated applications of hCOs as advanced in vitro models in early warning for DICT, drug metabolism-related cardiac safety assessment, and personalized drug evaluation. hCOs, human cardiac organoids; DICT, drug-induced cardiotoxicity; hPSCs, human pluripotent stem cells; DCM, dilated cardiomyopathy; MI, myocardial infarction.

With a number of advantages, hCOs have brought new transformation and developments to the preclinical drug evaluation. The evaluation has evolved from the traditional research on arrhythmia caused by a single electrophysiological interruption to a multi-dimensional system covering structure, function, and mechanism [117]. The construction of this multi-dimensional system offers more comprehensive and precise methods for cardiac safety assessment and is capable of conducting early warnings for DICT. Additionally, with the deep integration and innovation of cutting-edge technologies such as gene editing and OoC with hCOs, hCOs have shown broader application prospects. Three specific application areas of hCOs in preclinical drug evaluation are outlined in this section (Figure 3).

#### 4.2.1. Early Warning for DICT

Currently, hCOs are used to conduct multi-dimensional evaluations of drug toxicity to achieve early warning of DICT. This approach not only mitigates the risk of post-marketing drug withdrawal due to cardiac safety concerns but also enhances patient medication safety. In 2018, Archer et al. [42] used the 3D Cardiac Microtissue model for the first time to conduct a comprehensive assessment of various types of structurally cardiotoxic drugs. The results showed that combining imaging parameters with the assessment of cell viability in 3D Cardiac Microtissue could effectively predict the effect of structural cardiac toxins in vivo, with an overall sensitivity of 73% and a specificity of 86%. This discovery fully demonstrated that 3D Cardiac Microtissue was an effective model for evaluating drug-induced structural cardiotoxicity. Lee et al. [26] selected three representative drugs, nifedipine, E-4031, and flecainide, which target calcium, potassium, and sodium channels, respectively, and successfully verified the effectiveness of hCOs derived from hiPSCs in evaluating the electrophysiological effects of ion channel regulators. Subsequently, the preclinical early warning ability of the hCOs platform for DICT was analyzed in depth using five clinical drugs with clear cardiotoxicity, namely DOX, sorafenib, 5-fluorouracil (5-FU), vandetanib, and sparfloxacin. Finally, a comprehensive approach involving the integration of multi-electrode array (MEA) detection, viability detection, lactate dehydrogenase (LDH) toxicity determination, and contractility determination was employed to ascertain the non-toxic nature of Echinochrome A (EchA) concerning cell viability and cardiac function.

Cardiotoxicity has become a major cause of mortality among cancer patients [8]. Consequently, the employment of suitable in vitro models is imperative for the early prediction of cardiotoxicity associated with anti-cancer drugs. Beck et al. [118] used hCOs to conduct a direct cardiotoxicity assessment of trametinib, a drug for treating melanoma. After exposure to trametinib for six days, CMs showed atrophy and weakened contraction. Further research found that this cardiotoxicity is reversible, consistent with in vivo studies. Chen et al. [119] exposed hCOs to varying concentrations of the anti-cancer drug DOX. By detecting morphological changes, CMs’ viability, contractility, gene expression of apoptosis-related factors, release of clinical biomarkers of myocardial injury, abnormal expression of cardiac function markers, gene expression of inflammatory factors and pro-fibrotic factors, as well as changes in mitochondrial membrane potential, the DOX-induced cardiotoxicity was accurately characterized. In addition, another study [43] assessed the beating frequency, cell viability, cell apoptosis, as well as calcium handling capacity, revealing that vascularized and chambered cardiac organoids (vcCOs) produced a significant dose-dependent toxic response to DOX. These findings indicate that the hCO model is a reliable in vitro tool for preclinical DICT detection, opening up a new approach for the early prediction of cardiotoxicity of anti-cancer drugs.

In the process of drug development, understanding the toxicity of drugs to cardiac development can provide early warnings of their potential harm to cardiac embryonic development, providing a basis for the safety assessment of drug use in pregnant women. Hoang et al. [120] applied hCOs to study the effects of nine drugs in the FDA pregnancy classification system (covering the entire range of teratogenic risks from Class A (safe) to Class X (toxic)) on embryonic hearts. Exposure to thalidomide, a known teratogenic agent, was found to result in severe impairment of hCOs manifested by reduced cardiac tissue and morphological changes. In addition, the test results of three antibiotics (amoxicillin, rifampicin, and doxycycline) showed that the drug-induced toxicity to hCOs increased with the increase in teratogenicity risk. Mendjan’s team [56] constructed the multi-chamber hCOs platform and revealed that drugs such as thalidomide and retinoid derivatives could induce ventricle-specific defects. Volmert et al. [121] used hCOs to study the developmental toxicity of ondansetron (a commonly prescribed drug for preventing nausea and vomiting during pregnancy) on the heart. The study revealed that ondansetron can inhibit the differentiation and maturation of ventricular cardiomyocytes, which is consistent with epidemiological studies showing that ondansetron use during pregnancy increases the risk of ventricular septal defect. In addition, long-term use of ondansetron also had adverse effects on the electrophysiological maturation of the embryonic/fetal heart. These studies fully demonstrated the great potential of hCOs in evaluating drug-induced developmental toxicity.

#### 4.2.2. Drug Metabolism-Related Cardiac Safety Assessment

Many drugs are safe in their natural chemical composition, but their metabolites may have cardiotoxicity. Against this backdrop, the emerging Organoids-on-Chip technology, combining stem cell-derived organoids with advanced OoC technology, has provided a new platform for in vitro biomimetic microphysiological system construction [122]. Recently, two-organoid or multi-organoid devices with higher biological complexity (also known as “body-on-a-chip” systems) began to emerge, demonstrating potential far beyond that of a single organoid platform [123]. This “body-on-a-chip” system enables more comprehensive evaluation of drug toxicity and metabolic characteristics for specific organs, reducing false negatives [120] and thereby offering a more powerful tool for drug safety studies.

In 2017, Skardal’s team constructed a multi-organoids-on-a-chip system for liver, heart, and lung [124] to simulate the interactive nature of the human body and verify that the function of the heart depends on the metabolic capacity of the upstream liver. In 2020, this team developed a six-organoid integrated platform [125] that connected liver, cardiac, lung, endothelium, brain, and testes organoids. Prodrug capecitabine was found to be metabolized to 5-FU in liver organoids and to be toxic to hCOs, resulting in reduced cell viability. Yin et al. constructed a two-organoids-on-a-chip system [126] in 2021 to evaluate the cardiac safety of the antidepressant clomipramine. It has been found that clomipramine, which was metabolized to desmethylclomipramine by the CYP450 enzyme in liver organoids, significantly reduced the cell viability of hCOs, and also impairs cardiac contraction and calcium handling, indicating that the cardiotoxicity of clomipramine was related to hepatic metabolism. These achievements demonstrate the significant potential of the “body-on-a-chip” system in evaluating drug metabolism and toxicity. By overcoming the limitations of single hCO models in drug metabolic evaluation, this system provides a more physiologically relevant platform for cardiac safety assessment and opens up a new way for in vitro assessment of the efficacy and safety of drugs.

#### 4.2.3. Personalized Drug Evaluation

The organoid pathological model, constructed by integrating organoids and pathological models, has the potential to be used for high-throughput toxicity testing and to improve the accuracy of toxicity prediction. The employment of this model to simulate the complex interaction between diseases and drugs provides a reliable and accurate platform for studying drug mechanisms, which is conducive to improving the success rate of drug research and development, reducing adverse reactions, and promoting the development of precision medicine [31]. Mei’s team exposed hCOs to continuous adrenaline stimulation and hypoxia to simulate chronic myocardial infarction [85]. They used this model to confirm that DOX worsens MI-induced injury, including key mechanisms such as oxidative stress, DNA damage, and mitochondrial dysfunction. This study suggests hCO pathological models can assess DICT. During the COVID-19 pandemic, the team developed a viable COVID-19 infection model in hCOs [90] using IL-1β treatment to evaluate the efficacy and potential side effects of four common immunomodulatory drugs: IL-1 receptor antagonist (IL-1RA), Tocilizumab, Baricitinib, and Dexamethasone. This study found that although Dexamethasone could alleviate the contractile disorder of COVID-19-infected hCOs, it simultaneously induced thrombosis and fibrosis, revealing that its efficacy coexisted with cardiotoxicity. Furthermore, another study employed hCOs to model COVID-19-associated cardiac injury. Integrated transcriptomic analysis revealed that the inflammatory cytokine storm drives cardiac dysfunction via specific epigenetic pathways and further validated the therapeutic mechanism of BET inhibitors in this context [45]. A research team [94] has developed an hCO model based on hiPSCs to simulate diabetic cardiomyopathy and assess metformin’s safety and efficacy. The results demonstrated that metformin treatment attenuated adverse events induced by high-glucose and high-lipid conditions, providing supporting evidence for the potential use of this drug in the treatment of diabetic cardiomyopathy. This model not only demonstrates its utility in assessing the safety and efficacy of known drugs but also provides a platform for screening new potential therapeutic agents.

In addition to the disease-specific models mentioned above, hCOs can also construct patient-specific models to evaluate cardiotoxicity in specific patients, providing a key basis for clinical precision medicine and avoiding adverse reactions [59]. Buono et al. [72] found that hCOs derived from healthy individuals and those with genetic cardiomyopathy show significant differences in structure, morphology, and contractile function, indicating that the hCO model based on patient cells provided a platform closer to the actual situation of patients for drug evaluation. A recent study [70] constructed an ARVC hCO model using patient-derived hiPSCs and provided direct evidence that testosterone plays a profibrotic role in ARVC. These studies indicated that patient-derived hCOs could accurately capture individual differences in drug responses, which held great promise in preclinical drug evaluation and could significantly promote the development of precision medicine.

Owing to their unique advantages, hCOs play an increasingly important role in preclinical drug evaluation, significantly enhancing both drug development efficiency and clinical medication safety. Looking ahead, the reliable translation of in vitro findings from hCOs into clinically relevant insights represents the core challenge for assessing their practical value. Achieving this translation depends on a systematic validation strategy. First, it is essential to establish quantitative correlations between in vitro functional readouts from hCOs—such as changes in action potential or diminished contractile force—and clinical endpoints or biomarkers, such as QT interval prolongation or decreased left ventricular ejection fraction. Second, retrospective studies are needed to systematically analyze the concordance and discordance between hCOs’ predictions for drugs with known clinical toxicity profiles and real-world data, while objectively dissecting the inherent model limitations revealed by cases of inconsistency, such as metabolic immaturity or a lack of systemic interactions. Although currently, directly published cases in which hCOs have failed to predict specific clinical outcomes remain relatively limited—primarily because the technology has not yet been widely adopted as a primary decision-making tool in late-stage drug development—the field generally acknowledges these inherent limitations. Consequently, a pragmatic translation pathway is to position hCOs not as an isolated “arbiter,” but rather as a powerful component within a “weight-of-evidence” framework, complementing other non-clinical data and early clinical biomarkers to systematically reduce drug development risks.

## 5. Challenge and Outlook

At present, the hCO model has made certain progress in the construction of pathological models and preclinical drug evaluation, but it still faces many challenges. Among them, insufficient maturity constitutes the core limitation. Current hCOs retain the core structural, electrophysiological, and metabolic characteristics of the fetal heart and have not yet fully transitioned to the physiological state of the adult heart. This fundamentally limits their ability to accurately simulate adult cardiac function and predict drug responses [127]. This limitation is manifested in the following dimensions. (1) Predictive bias due to electrophysiological immaturity: The characteristic action potential features of the embryonic heart, such as slow upstroke and shortened plateau phase, along with the differential expression of key ion channels [128], are retained in hCOs with insufficient maturity. This results in an inherent bias in pharmacological assessment: the model may amplify the proarrhythmic risk dominated by hERG channel blockade, yet struggle to accurately capture drug effects that induce toxicity by disrupting calcium homeostasis. (2) Altered toxicological response resulting from metabolic profile differences: Concurrently, the metabolic profile of hCOs more closely resembles that of the fetal heart, relying predominantly on glycolysis and lacking the efficient energy production system of adult myocardium, which depends on mitochondrial fatty acid β-oxidation (contributing 70–90% of ATP) [129]. This metabolic immaturity likely renders the model less sensitive to drugs targeting mitochondrial function or fatty acid metabolism (such as certain chemotherapeutic agents) and unable to faithfully simulate the pathophysiological responses of the heart under energy stress conditions. (3) Structural and microenvironmental oversimplification: Current models lack functional vascular networks, immune cells, and neural innervation, and thus cannot replicate the complex pathophysiological processes involving inter-organ interactions and systemic-level regulation [121].

Meanwhile, while recent advances have enabled the construction of vascularized hCOs [43,51,130], significant challenges remain in achieving functional perfusion and clinically relevant vascular exchange. Key limitations include: (1) Perfusion systems often lack physiologically relevant hemodynamic stimuli such as shear stress and pulsatile pressure, which restrict endothelial maturation and functional barrier formation. (2) The generated vascular networks are structurally simplified, lacking hierarchical organization (arteriole-capillary-venule) and stable coverage by pericytes/smooth muscle cells, resulting in compromised vessel integrity and impaired flow regulation. (3) Substance-exchange fidelity is insufficient to mimic the complex kinetics of trans-endothelial transport in vivo (e.g., oxygen gradients, drug diffusion, and active transport), particularly due to the absence of a functional blood-tissue barrier, thereby limiting accurate prediction of drug distribution and metabolite clearance. (4) As isolated in vitro systems, current vascularized hCOs cannot dynamically integrate with immune modulation, neural innervation, or systemic metabolic feedback—interactions that are critical mechanisms underlying DICT.

Additionally, the standardization and repeatability of hCOs are pivotal considerations. The differences among laboratories in terms of cell types, cytokine concentrations, extracellular matrix scaffold types, mechanical stimulation, the time and methods of hiPSC induction, lead to significant batch effects in organoids, affecting the repeatability of the model [131,132]. This situation has resulted in a lack of comparability between datasets from different laboratories, thereby hindering regulatory acceptance. Moreover, it is well established that the pharmacological effects of drugs in vivo depend not only on their direct actions on the heart but also on integrated physiological processes such as hepatic metabolic transformation, renal clearance efficiency, plasma protein binding, and neuroendocrine regulation. Single hCO models are unable to recapitulate this complex multi-organ interactive environment [101]. Particularly for prodrugs, their potential cardiotoxicity often relies on the bioactivation into reactive intermediates via hepatic metabolism—a critical conversion process that cannot be reproduced in isolated hCO systems. Finally, the obstacles to integrating multi-dimensional assessment data and clinical transformation are equally significant. The heterogeneity of multi-dimensional evaluation data, such as structure, cell composition, function, and molecule, increases the complexity of integrated analysis and affects the precise interpretation of drug responses. Conversely, the absence of an international certification framework engenders suboptimal comparability of cross-platform data. Despite the evident success of organoids in a laboratory setting, the integration of these models with clinical practice remains an urgent scientific challenge that must be addressed.

One of the key directions for future research is to enhance the maturity of hCOs. At present, researchers have developed a variety of strategies, such as extending the culture time, electrical stimulation, mechanical stretching, adding specific growth factors [28], activating the AMPK/ERR signaling axis [133], or co-culture with non-cardiac cells [134], which have effectively enhanced the biological relevance of the hCO model. Moreover, to advance the vascularization of hCOs, future progress will require combining dynamic perfusion engineering, multicellular co-culture systems, and the establishment of quantitative vascular-function metrics (e.g., permeability, perfusion efficiency, oxygen gradient) to advance these models from structural mimicry toward functional equivalence [135]. Microfluidic systems enable the simulation of dynamic inter-organ interactions, providing a platform for drug evaluation under near-physiological conditions [28]. Existing multi-organ-on-a-chip can model functional crosstalk between the heart and other organs, yet remain oversimplified in replicating complex in vivo metabolic and immune responses. Future efforts should focus on enhancing the physiological complexity and integration of these models, combined with validation using traditional preclinical approaches, to improve their predictive and translational value in drug development. Driven by continuous progress in manufacturing technology, sensing materials [15], and AI algorithms [136], interdisciplinary collaboration and regulatory engagement are expected to enable high-throughput, standardized, and automated production of organoids. These advancements will further facilitate intelligent monitoring, evaluation, and control of organoid models [31], thereby enhancing their critical role in precise drug screening and precision medicine. Recently, a study [95] developed an integrated AI and image analysis approach for rapid quantitative assessment of hCO inflammation, allowing high-precision, non-invasive, and dynamic monitoring of inflammatory levels. The team also developed a novel treatment strategy targeting inflammation-induced metabolic dysfunction, which effectively improved metabolic homeostasis in organoid models. This work offers new avenues for drug screening and precision medicine in cardiovascular diseases. These new technologies and methods promote the preclinical drug evaluation to develop in a more personalized and dynamic direction, laying a solid foundation for global drug research and development.

## 6. Conclusions

This review provides a comprehensive overview of the rapid advancement of hCO technology and its increasingly vital role in preclinical drug evaluation. Unlike previous reviews, this paper goes beyond merely describing construction techniques. Instead, it presents a unique integrative perspective, systematically constructing a clear knowledge framework that spans from “direct assembly and self-assembly strategies” to “three major approaches for constructing pathological models,” ultimately extending to “multidimensional drug evaluation applications.” This framework powerfully demonstrates the immense potential of hCOs to overcome limitations of traditional models and deliver human-specific data.

Crucially, we highlight that the application of hCOs has received critical support from a revolutionary shift in regulatory science worldwide, laying a solid foundation for their transition from research tools to regulatory-accepted evaluation standards. Despite the persistent challenges in maturity, vascularization, standardization and reproducibility, and the integration of multi-dimensional data, hCOs hold promise for significantly advancing CVDs modeling, drug safety assessment, and precision medicine through integration with cutting-edge technologies such as OoC, multi-omics analysis, and AI. In summary, this review provides a systematic and forward-looking framework to elucidate how hCOs serve as a pivotal bridge connecting basic research, drug development, and clinical translation, laying the cornerstone for establishing a more efficient and predictable paradigm in future drug discovery.

## Figures and Tables

**Table 1:** Summary of recent reviews on hCOs and drug discovery (2021–2025).

Year	Focus Areas	Content Outline	References
2025	Different hCO construction techniques and their applications in disease modeling.	Three primary techniques for constructing hCOs: hEHTs, self-organizing organoids, and OoC technology; Applications of hCOs in disease; Applications of multi-organoid models; hCOs assessment techniques.	[27]
2025	Interactions between the heart and other organs, particularly the role of hiPSC-derived organoid models in heart-brain, heart-kidney, and heart-liver interactions in CVDs.	Application of 2D co-culture models in studying cell–cell interactions; Human organoid models for studying inter-organ communication; Application of human organoid models; The maturation of cardiomyocytes in hiPSC-derived cardiac models; Omics tools for the integrative study of inter-organ communications.	[32]
2025	Advances in the use of hPSC-derived hCOs for modeling cardiac development, CVDs, and drug cardiotoxicity research.	3D modeling of the heart; hCOs for modeling human cardiogenesis; hCOs for disease modeling; hCOs for examining cardiotoxicity and drug discovery; Methods for assessing hCOs morphology and function.	[30]
2024	Broad overview of organoid applications in all fields.	History of organoid development; Developments and advances in various organoids; Applications in disease modeling, drug discovery and toxicity assessment, precision medicine, and regenerative medicine; Challenges and prospects.	[31]
2023	Progress in the application of hPSC-derived hCOs and heart-on-chip in anti-cancer drug-induced cardiotoxicity.	Current status of anti-cancer drug cardiotoxicity; hiPSC-CMs and 2D system to study anti-cancer therapy-induced cardiotoxicity; 3D cardiac constructs in cardiotoxicity research; heart-on-chip in anti-cancer drug-induced cardiotoxicity research.	[8]
2022	Technological advances in several cardiovascular models.	Latest advances in the directed stem cell differentiation approaches; Recent progress in the development of several cardiovascular models, such as hCOs, microtissues, hEHTs, and microphysiological systems; Discussion on how to define the context for the use of currently available cardiac tissue models.	[28]
2021	Progress in novel hCOs generation methods and their advantages and disadvantages.	Self-organizing vs. directed assembly organoid technologies; The advantages and disadvantages of each approach; Their translational applications for advancing cardiovascular studies and the treatment of heart disorders.	[29]

hCOs, human cardiac organoids; hEHTs, human engineered heart tissues; OoC, organ-on-a-Chip; CVDs, cardiovascular diseases; hiPSC-CMs, hiPSC-derived cardiomyocytes.

**Table 3:** Comparison of Pathological Modeling Strategies for hCOs.

	Patient-Specific	Gene-Edited	Microenvironment-Modulated
Reproducibility	Relatively low (due to significant inter-individual genetic background differences)	High (isogenic phenotype with controllable genetic background)	Moderate (depending on the level of standardization in stimulation conditions)
Translational Relevance	High (directly reflects individual patient disease phenotype and drug response)	Moderate (establishes genotype-phenotype causality for monogenic diseases but limited for polygenic/sporadic cases)	Variable (highly relevant for environmentally driven acquired diseases, limited for hereditary conditions)
Cost and Scalability	High cost, low scalability	High initial cost, moderate scalability in later stages	Relatively low cost, high scalability
Suitability for Specific Diseases	Hereditary cardiomyopathy and arrhythmias, rare diseases	Monogenic hereditary heart diseases, such as HCM and DCM	Acquired heart diseases, such as MI, Diabetic cardiomyopathy, drug-induced myocardial injury; multifactorial complex diseases
Research applicability	In-depth mechanism research or precision medicine case studies	Moderate-throughput mechanistic studies or target validation.	Facilitates high-throughput drug screening or toxicity testing.

HCM, hypertrophic cardiomyopathy; DCM, dilated cardiomyopathy; MI, myocardial infarction.

## Data Availability

No new data were created or analyzed in this study. Data sharing is not applicable to this article.

## Abbreviations

hCOs	human cardiac organoids
DICT	drug-induced cardiotoxicity
CMs	cardiomyocytes
CVDs	cardiovascular diseases
APD	action potential duration
hPSCs	human pluripotent stem cells
hESCs	human embryonic stem cells
hiPSCs	human induced pluripotent stem cells
hEHTs	human engineered heart tissues
hPSC-CMs	hPSC-derived cardiomyocytes
HFOs	heart-forming organoids
vcCOs	vascularized and chambered cardiac organoids
CHD	congenital heart disease
MI	myocardial infarction
HCM	hypertrophic cardiomyopathy
DCM	dilated cardiomyopathy
DMD	Duchenne muscular dystrophy
DOX	doxorubicin
OoC	organ-on-a-Chip
NAMs	New Approach Methodologies
